# Augmentation of the heat shock axis during exceptional longevity in Ames dwarf mice

**DOI:** 10.1007/s11357-021-00362-w

**Published:** 2021-04-13

**Authors:** Rachana Trivedi, Bailey Knopf, Jitendra Kumar Tripathi, Shar Rakoczy, Gunjan D. Manocha, Holly Brown-Borg, Donald A. Jurivich

**Affiliations:** 1 Department of Geriatrics, School of Medicine and Health Sciences, University of North Dakota, Grand Forks, ND 58202 USA; 2 Department of Biomedical Sciences, School of Medicine and Health Sciences, University of North Dakota, Grand Forks, ND 58202 USA

**Keywords:** Heat shock, Aging, Longevity, HSF, Ames dwarf

## Abstract

How the heat shock axis, repair pathways, and proteostasis impact the rate of aging is not fully understood. Recent reports indicate that normal aging leads to a 50% change in several regulatory elements of the heat shock axis. Most notably is the age-dependent enhancement of inhibitory signals associated with accumulated heat shock proteins and hyper-acetylation associated with marked attenuation of heat shock factor 1 (HSF1)–DNA binding activity. Because exceptional longevity is associated with increased resistance to stress, this study evaluated regulatory check points of the heat shock axis in liver extracts from 12 months and 24 months long-lived Ames dwarf mice and compared these findings with aging wild-type mice. This analysis showed that 12M dwarf and wild-type mice have comparable stress responses, whereas old dwarf mice, unlike old wild-type mice, preserve and enhance activating elements of the heat shock axis. Old dwarf mice thwart negative regulation of the heat shock axis typically observed in usual aging such as noted in HSF1 phosphorylation at Ser307 residue, acetylation within its DNA binding domain, and reduction in proteins that attenuate HSF1–DNA binding. Unlike usual aging, dwarf HSF1 protein and mRNA levels increase with age and further enhance by stress. Together these observations suggest that exceptional longevity is associated with compensatory and enhanced HSF1 regulation as an adaptation to age-dependent forces that otherwise downregulate the heat shock axis.

## Introduction

Aging and attenuated stress responses are commonly observed in different organisms ranging from yeast to man [1–3]. Understanding the molecular mechanism involved in sensing stress and activating the stress response axis is essential for studying the aging process. HSF1 is the master transcription regulator of heat shock responses (HSR) that transcriptionally activates the proteostasis network [4–7]. The role of HSF1 in exceptional longevity, usual aging, and the proteostasis network mostly is documented in lower, poikilothermic organisms such as *C. elegans* [8–10]. HSF1 is subject to feedback inhibition through interaction with heat shock proteins (HSPs), HBP1, and by direct acetylation [11–14]. Deacetylase activity of sirtuin 1 protein (SIRT1) and other deacetylases facilitate HSF1 binding to a DNA sequence consisting of inverted repeats of nGAAn known as heat shock element (HSE) [15–17]. In addition to heat shock, treatment of cells with heavy metals, uncouplers of oxidative phosphorylation, H_2_O_2_, non-steroidal anti-inflammatory drugs and alcohol, as well as infection and inflammation promote HSF1 trimerization and its DNA-binding activity [18, 19]. The diminished ability of HSF1 to bind DNA in cells and tissue from aging humans and mammals as well as the inability of the human brain to counter proteotoxic stress during age-related neurodegeneration support the hypothesis that aging profoundly compromises the HSR [20–23].

To better understand age-dependent defects in HSR, multiple regulatory elements driving HSF1 from an inert to active state need to be assessed. Under normal stress inducing conditions, HSF1 goes through various post-translational modifications such as phosphorylation and deacetylation [16, 24–26]. Monomeric HSF1 interacts with HSP90, HSP70, and HSP40, and these molecular brakes render HSF1 transcriptionally inactive during non-stress conditions and during the attenuation phase of the HSR. Thus, tight regulation of activation, initiation, execution, and termination of the HSR occurs through complex post-translational modifications and protein–protein interactions [27–31]. With these different regulatory steps of HSF1 in mind, this study uses both in vivo and *ex vivo* (heat shock) experiments with liver tissue to examine if exceptionally long lived mice regulate HSF1 any differently than wild-type mice during the aging process. The principal hypothesis of this study was that exceptionally long-living mice generate a highly enhanced HSR axis that declines with age less readily than wild-type mice. However, the data suggest a very different hypothesis, namely, that exceptional longevity enhances rather than preserves the HSR in these long-lived mice age. *Ex vivo* liver experiments further amplify the thesis that exceptional longevity triggers a highly adaptive, if not overly compensated, stress response. Thus, the increase in longevity reported for Ames dwarf mice is in fact correlated with increased resistance to heat stress. Taken together, enhanced stress resistance may be an essential function in Ames mice that leads to their increased longevity.

## Material and methods

### Antibodies and reagents

Anti-HSF1 (51034-1-AP), anti-HSP90 (13171-1-AP), anti-HSP70 (10995-1-AP), anti-HSP40 (13174-1-AP), and anti-GAPDH (10494-1-AP) were all purchased from ProteinTech Group while anti-HSF2 (ab126252), anti-HSF1 ser307 (ab75905), and anti-HSF1 ser326 (ab115702) were purchased from Abcam Inc. Anti-SIRT1 was purchased from Cell signaling technology while anti-HSBP1 was purchased from MyBiosource. The horseradish peroxidase–conjugated secondary antibodies were purchased from BioRad Laboratories Inc. Real-time PCR primers for HSF1 (F-ACAGTGTCACCCGGCTGTTG and R-GACTGCACCAGTGAGATGAGGAA), HSF2 (F-GCAGTGTTGTTCAACATGTGTCAG and R-AGTTCCCATCCAGGAATGCAAG), and endogenous control B2M (F-ATGGGAAGCCGAAC-ATACTG and R-CAGTCTCAGTGGGGGTGAAT) were purchased from Eurofins MWG Genomics LLC and HSE probe for electrophoretic mobility shift assay (EMSA) was purchased from Integrated DNA Technologies. Anti-acetylated lysine (9441) was purchased from Cell Signaling Technology while anti-rabbit IgG HRP (5220-0337) was purchased from Sera Care.

### Animals

Male and female Ames dwarf (12 and 24 months) mice were bred and maintained at the Center for Biomedical Research, University of North Dakota, under controlled pathogen-free conditions of photoperiod 12:12-h light/dark cycles and temperature (22±1 °C) with ad libitum access to food, water and exercise. These mice have a heterogeneous genetic background from a colony that has been closed for over 30 years. The investigation conforms to the National Research Council of the National Academies Guide for the Care and Use of Laboratory Animals (8th edition) and was reviewed and approved by the UND IACUC. Ames dwarf mice aged 12 and 24 months were sacrificed by in-cage CO_2_ asphyxiation followed by de-capitation. The liver tissue from 5 to 8 mice per group was isolated and lysed using RIPA buffer, and protein was quantified using the Bradford method.

### EMSA

EMSAs were performed using the LightShift Chemiluminescent EMSA Kit (Thermo Scientific) according to the manufacturer’s protocol as described [30]. Whole cell extracts from liver tissue were prepared in buffer C (20mM HEPES, pH 7.9, 25% glycerol, 0.42M NaCl, 1.5 mM MgCl_2_, 0.2 mM EDTA, 0.5 mM PMSF, and 0.5 mM DTT), and 15 μg protein was incubated with 1 μg poly[d(I-C)], 2 μl of 10× binding buffer, 1 μl of 50% glycerol, 1 μl of 1% NP-40, 1 μl of 100 mM MgCl_2_ and 20 fmol of biotin end-labeled probe (HSE biotinylated probe CTAGAAGCTTCTAGAAGCTTCTAG) in a total volume of 20 μl and incubated for 30 min at 25 °C. After separation by 5% TBE polyacrylamide gel electrophoresis, the protein–DNA complexes were transferred to a nylon membrane (Pierce) and the membranes were UV-cross linked for 10 min. The biotin-labeled DNA was probed with streptavidin-HRP conjugate for chemiluminescence detection. The band corresponding to the HSF1–HSE complex was quantitated for optical density measurement and plotted ± SEM (*n* = 3–6).

### 2D gel electrophoresis

2D gel electrophoresis was performed by Kendrick’s Labs Inc. (Madison, Wisconsin) according to the carrier ampholine method of isoelectric focusing. Isoelectric focusing was carried out in a glass tube of inner diameter 3.3 mm using 2.0% pH 3–10 Isodalt mix Servalytes (Serva, Heidelberg, Germany) for 20,000 volt-hrs. One microgram of an IEF internal standard, tropomyosin, was added to each sample. This protein migrates as a doublet with lower polypeptide spot of MW 33,000 and pI 5.2. The enclosed tube gel pH gradient plot for this set of Servalytes was determined with a surface pH electrode.

After equilibration for 10 min in Buffer “O” (10% glycerol, 50 mM dithiothreitol, 2.3% SDS, and 0.0625 M tris, pH 6.8), each tube gel was sealed to the top of a stacking gel that overlaid a 10% acrylamide slab gel (1.00 mm thick). SDS slab gel electrophoresis was carried out for about 5 h at 25 mA/gel. After slab gel electrophoresis, the gels were placed in transfer buffer (10 mM CAPS, pH 11.0, 10% MeOH) and transblotted onto PVDF membranes overnight at 225 mA and approximately 100 volts/two gels. The following proteins (Millipore, Sigma) were used as molecular weight standards: myosin (220,000), phosphorylase A (94,000), catalase (60,000), actin (43,000), carbonic anhydrase (29,000), and lysozyme (14,000). These standards appear as bands at the basic edge of the Coomassie Brilliant Blue R-250-stained membrane.

The blots were stained with Coomassie Brilliant Blue R-250 and desktop scanned. The blots were wetted in 100% methanol, rinsed briefly in Tween-20 tris-buffered saline (TTBS) and blocked for 2 h in 5% bovine serum albumin (BSA) in TTBS. The blots were then incubated in primary antibody (anti-acetylated lysine [Cell Signaling, Cat # 9441, Lot # 11] diluted 1:10,000 in 2% BSA in TTBS) overnight and rinsed 3 × 10 min in TTBS. The blots were then placed in secondary antibody (anti-rabbit IgG HRP [Sera Care, Cat #5220-0337, Lot #10245261] diluted 1:20,000 in 2% BSA in TTBS) for 2 h, rinsed in TTBS as above, treated with ECL, and exposed to X-ray film.

### Western blotting

Liver lysates (20 μg protein) were resolved by SDS-PAGE and transferred to polyvinylidene difluoride membranes for Western blotting using anti-HSF1 (1:1500), anti-HSF2 (1:800), anti-HSP40 (1:2000), anti-HSP70 (1:2000), anti-HSP90 (1:2000), anti-HSF1 ser307 (1:500), anti-HSF1 ser326 (1:500), anti-SIRT1 (1:1000), anti-HSBP1 (1:1000) and anti-GAPDH (1:40,000; loading control) antibodies. Antibody binding was detected using enhanced chemiluminescence for detection (Omega Lum G Aplegen). Western blots were quantified using UltraQuant software for densitometry. Optical density (O.D.) of bands was normalized against their respective loading controls and averaged (±SEM).

### Real-time PCR

RNA was extracted from the liver tissue of middle-aged and old (12 months and 24 months) Ames dwarf mice using AutoGen’s Quick Gene RNA kit. Real time PCR for HSF1 and HSF2 mRNA with the housekeeping gene, Beta 2 microglobulin (B2M), was performed with 3μl of a 1:10 dilution of the neat RNA using One-step RT-PCR Bio-Rad kit and the Bio-Rad CFX Connect System. Fold change in mRNA expression is represented as 2^ΔΔ−Ct^ ± SEM (*n*=10–14).

### Heat shock experiments

Male and female Ames dwarf type mice (12 months and 24 months) were sacrificed by CO_2_ asphyxiation, liver tissue was dissected and either flash frozen in liquid N_2_ or thin sliced and placed in ice-chilled, O_2_-infused, phosphate-buffered saline (PBS) prior to *ex vivo* heat shock experiments. For heat shock experiments, liver slices were transferred to pre-warmed, O_2_ infused, PBS at 37 °C or 43 °C for 1 h and then flash frozen for further analysis.

### Statistical analysis

Data are presented as bar graphs which represent the mean ± SEM. Values statistically different from controls were determined using one-way ANOVA (or two-way ANOVA where required). The Tukey–Kramer multiple comparisons post-test was used to determine *p* values.

## Results

### Changes in HSF1 and heat shock protein levels during aging of Ames dwarf mice

**Fig. 1:**
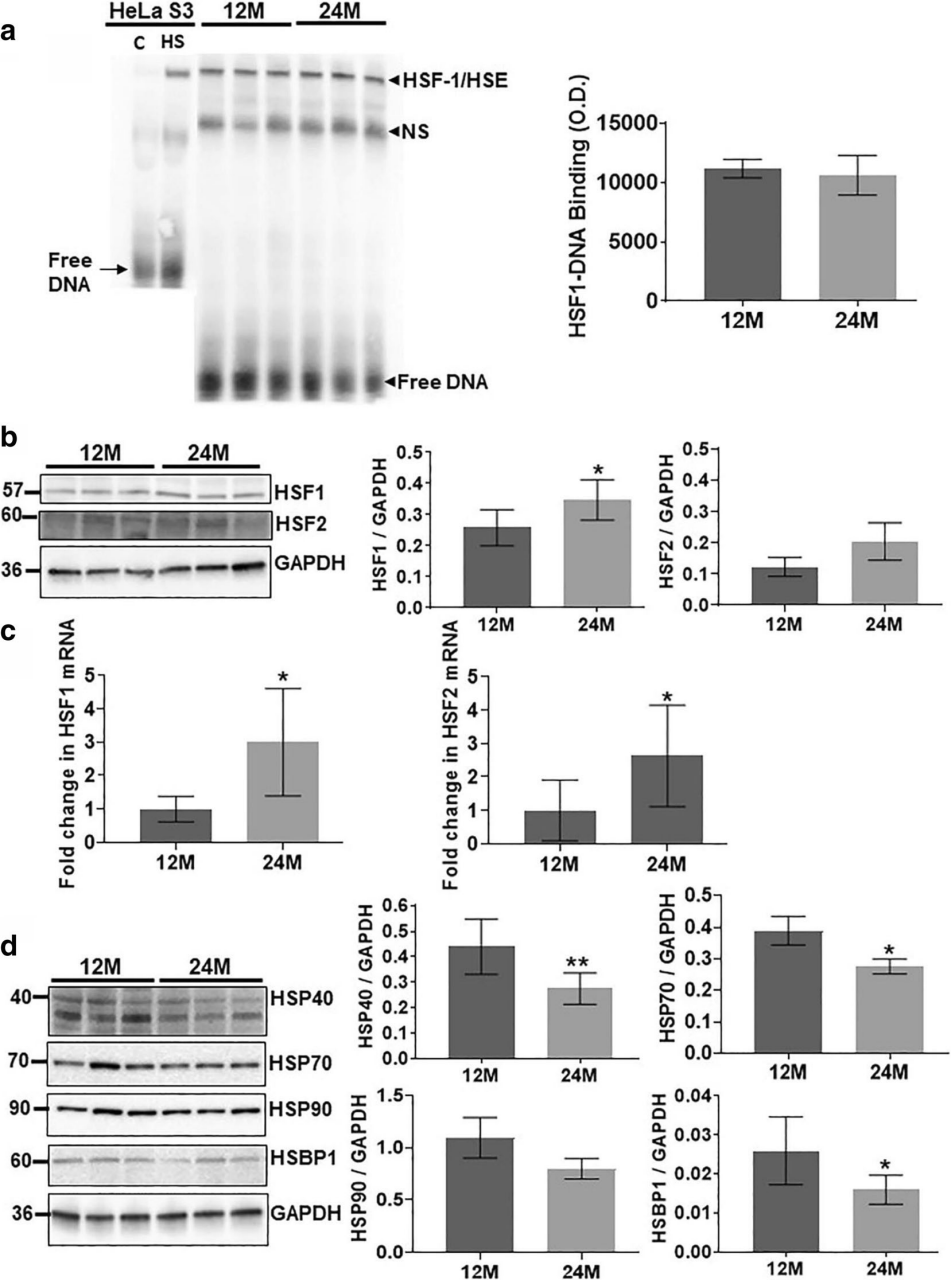
Changes in HSF1 and heat shock protein levels during aging of Ames dwarf mice. Middle age (12M) and old age (24M) Ames dwarf mouse liver tissue are compared**. a** An electromobility shift assay (EMSA) for HSF1–HSE binding is displayed which firstly shows a reference sample of 37 °C and 42 °C heat shocked HeLa S3 cells to demonstrate the specificity of stress inducible HSF1–DNA binding activity (first two lanes). Lanes 3–8 show representative samples of HSF1–HSE binding in liver extracts from middle age (12M) and old age mice (24M). The change in HSF1–HSE band was quantitated by optical density measurement and shows a 10% age-dependent decline in HSF1–HSE binding activity which was not statistically significant (*p = 0.6638) (*n* = 6). **b** Western blots and bar graphs of liver tissue extract from middle aged (12M) and old mice (24M) are shown for HSF1 and HSF2 protein. Protein levels for HSF1 (*n* = 8 and *p = 0.0116) and HSF2 (*n* = 8 and *p = 0.0505) increases with age in dwarf mouse liver. **c** Bar graphs display HSF1 and HSF2 mRNA levels analyzed by real-time polymerase chain reaction. A significant increase in HSF1 mRNA (*n* = 6 and *p = 0.0429) and HSF2 mRNA are associated with aging Ames dwarf mice (*n* = 6 and *p = 0.0471). **d** Western blot and bar graph analysis of age-dependent changes in mouse liver of putative regulators of HSF1 (Hsps and HSBP1) suggest that age leads to an overall decrease in heat shock protein levels with statistically significant changes observed with Hsp40 (*p = 0.0090) and Hsp70 protein (*p = .0455) levels. Another HSF1 inhibitory protein HSBP1 also declined with age (*p = 0.0161). (Abbreviation key: HSE, heat shock element; HSF, heat shock factor; NS, non-specific; O.D., optical density; WCE, whole cell extract, M, months, HS, heat shocked

Given that normal aging results in a 50% or greater reduction in HSF1–DNA binding activity [30], we firstly assessed HSF1–HSE binding activity in freshly isolated liver tissue from 12M and 24M old Ames dwarf mice. Figure 1 a shows a representative electromobility shift assay (EMSA) of HSF1 binding to an idealized HSE probe. Data from scanning densitometry reveals a 10% age-dependent decline in HSF1–DNA binding when 24M liver samples are compared to 12M (*n*=6) old liver tissue. This change was not statistically significant (*p*=0.2327). Thus, HSF1–DNA binding in dwarf mice does not decline with age as reported in Ames wild-type mice [30].

Because previous reports indicate age-dependent declines in HSF1 but not HSF2 protein levels [30, 32], both HSF1 and HSF2 protein and their mRNA expression levels were measured in 12M and 24M dwarf mice liver samples. Figure 1 b demonstrates Western blot analysis of HSF1 and HSF2 relative to GAPDH. Values from scanning densitometry presented in a bar graph show an age-dependent increase in HSF1 protein in 24M dwarf liver by 35% relative to 12M liver samples (*p* = 0.0032). HSF2 protein levels between 12M and 24M liver samples also showed slightly increased levels with age with borderline statistical insignificant (*p* = 0.0505).

To investigate whether the age-dependent increase in HSF1 protein levels noted in Ames dwarf mice was reflected at the mRNA level, real-time PCR was performed and Fig. 1c shows that HSF1 mRNA level increased over 3-fold (*p* = 0.0429). In a similar fashion, HSF2 mRNA levels increased over 2.5-fold with age (*p* = 0.0471).

Aging typically leads to increased heat shock protein levels that can interact with HSF1 and further negatively regulate it [33, 34]; thus, protein levels of three key HSF1-regulating heat shock proteins (HSP40, HSP70, and HSP90) were evaluated in Ames dwarf mice by Western blot analyses. Figure 1 d shows that HSP40, HSP70, and HSP90 protein levels decline with age in Ames dwarf mice. Bar graphs demonstrate age-dependent changes in HSP40, HSP70, and HSP90 protein levels (*p* = 0.0090, *p* = 0.0455, *p* = 0.2064, respectively).

Another negative regulator of HSF1 is the heat shock factor binding protein 1 (HSBP1) which is a nuclear protein and contains two extended arrays of hydrophobic repeats that interact with heptad repeats of HSF1. It inhibits HSF1 transcriptional activity by interfering with HSF1 trimerization [35, 36]. Thus, to investigate whether HSBP1 protein levels change in aging dwarf mice, we performed Western blot analysis using 12M and 24M liver lysates. Figure 1 d indicates that HSBP1 expression is decreased in old compared to middle age liver tissue from Ames dwarf mice. Thus, both protein–protein regulators of HSF1, HSPs and HSBP1, are attenuated with age in dwarf mice.

### Age-dependent posttranslational modification of HSF1

**Fig. 2:**
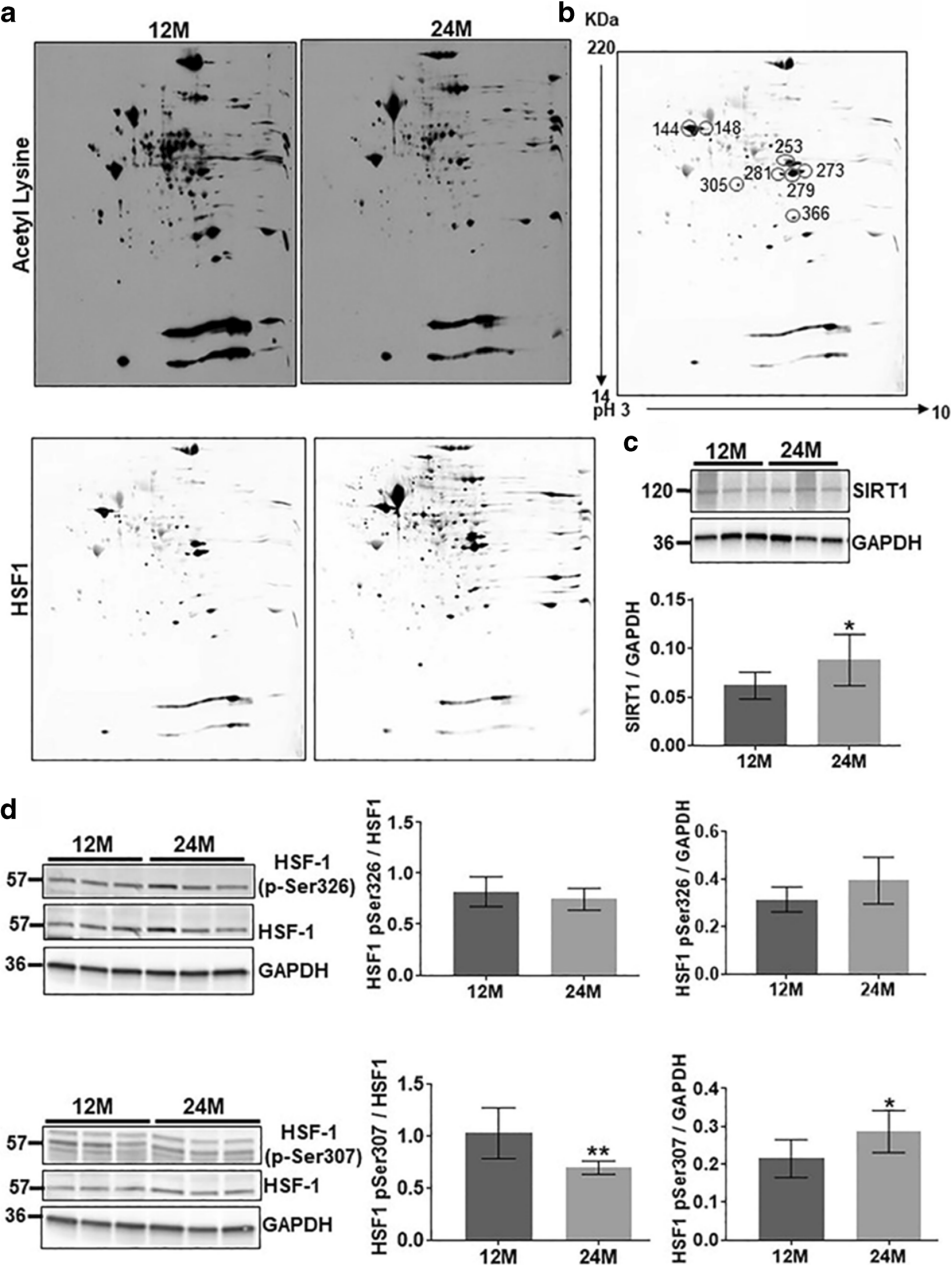
Age-dependent post-translational modification of HSF1. **a** A representative western blot analyses of two-dimensional (2D) gel electrophoresis show separated acetylated proteins and HSF1 from 12M and 24M mouse liver. **b **2D gel isofocused spots identified as acetylated HSF1 are highlighted and numbered. Age-dependent differences in HSF1 acetylation levels are listed in Table 1. **c** Western blot and bar graph analysis and quantification of SIRT1 level in middle aged (12M) and old age (24M) liver tissue. A 36% age-dependent increase in SIRT1 protein levels are found in aging Ames dwarf mice (*p*=.0259). **d** Western blot and densitometry bar graph analysis of HSF1 phosphorylation are shown with age. HSF1 phosphorylation at serine residues Ser307 (inhibitory) and Ser326 (stimulatory) shows that with normalization to either GAPDH or HSF1 protein levels that “inhibitory” phosphoserine 307 levels decline with age by 40% (*p = 0.0097), whereas the “activating” phosphoserine 326 levels do not change.

HSF1 transcriptional activity is mediated by diverse post-translational modifications, including phosphorylation and acetylation [24–26]. Knowing that acetylation of HSF1–DNA binding domain reduces HSF1–DNA binding activity [37, 38], we carried out two-dimensional gel electrophoresis followed by western blot analysis of whole cell lysate protein for total protein and HSF1-specific acetylation. Figure 2 a shows overall protein acetylation in 12M and 24M liver samples. It demonstrates that lysine acetylation varies among several isoelectric variants in aging dwarf mouse liver with several species undergoing both decreasing and increasing acetylation levels. Figure 2 b demonstrates HSF1 acetylated residues from a secondary Western blot of the two-dimensional gel electrophoresis. The results of scanning densitometry are summarized in Table 1. Three of the five HSF1 protein isoforms show 1.4- to 6-fold decrease in acetylation levels with age in dwarf mice. This finding is the opposite of what was reported in aging wild-type mice [30].

**Table 1:** Age-dependent differences in HSF1 acetylation levels in Ames dwarf mice

Spot#	HSF-1 probe		Spot#	Acetylation probe	
	12M	24M		12M	24M
253	1	1.45	253	1	−2.8
279	1	1.22	279	1	1.26
281	1	2.07	281	1	1.26
305	1	2.64	305	1	−1.4
366	1	2.9	366	1	−6

Given that protein and HSF1 acetylation status is tightly regulated by SIRT1, a nicotinamide adenosine dinucleotide (NAD)–dependent deacetylase, and that this enzymatic activity reportedly declines with age [39–42], we evaluated whether age alters the SIRT1 levels in 12M and 24M dwarf mice. Figure 2 c shows a Western blot of SIRT1 protein levels and a bar graph of densitometry values. Unlike wild type aging (data not shown), 24M dwarf liver tissue exhibits increased levels of SIRT1 protein levels.

As HSF1 exhibits both constitutive and inducible phosphorylation [24, 43, 44], we evaluated both activating and deactivating HSF1 phosphorylation changes with age in long-lived Ames dwarf mice. Firstly, we examined HSF1 phosphorylation on Ser326 which plays a key role in the induction of HSF1 transcriptional competency. HSF1 phosphorylation at Ser326 showed an increasing trend in 24M liver compared to 12M samples (Fig. 2d). By contrast, an age-dependent decrease was observed in phosphorylation levels of the HSF1-deactivating pSer307 when phosphoserine values were normalized against HSF1 (*p* = 0.0097); however, when normalized against GAPDH, HSF1 pSer307 was significantly increased (*p*=0.0168).

### *Ex vivo* heat shock response in middle age compared to old Ames dwarf mice

**Fig. 3:**
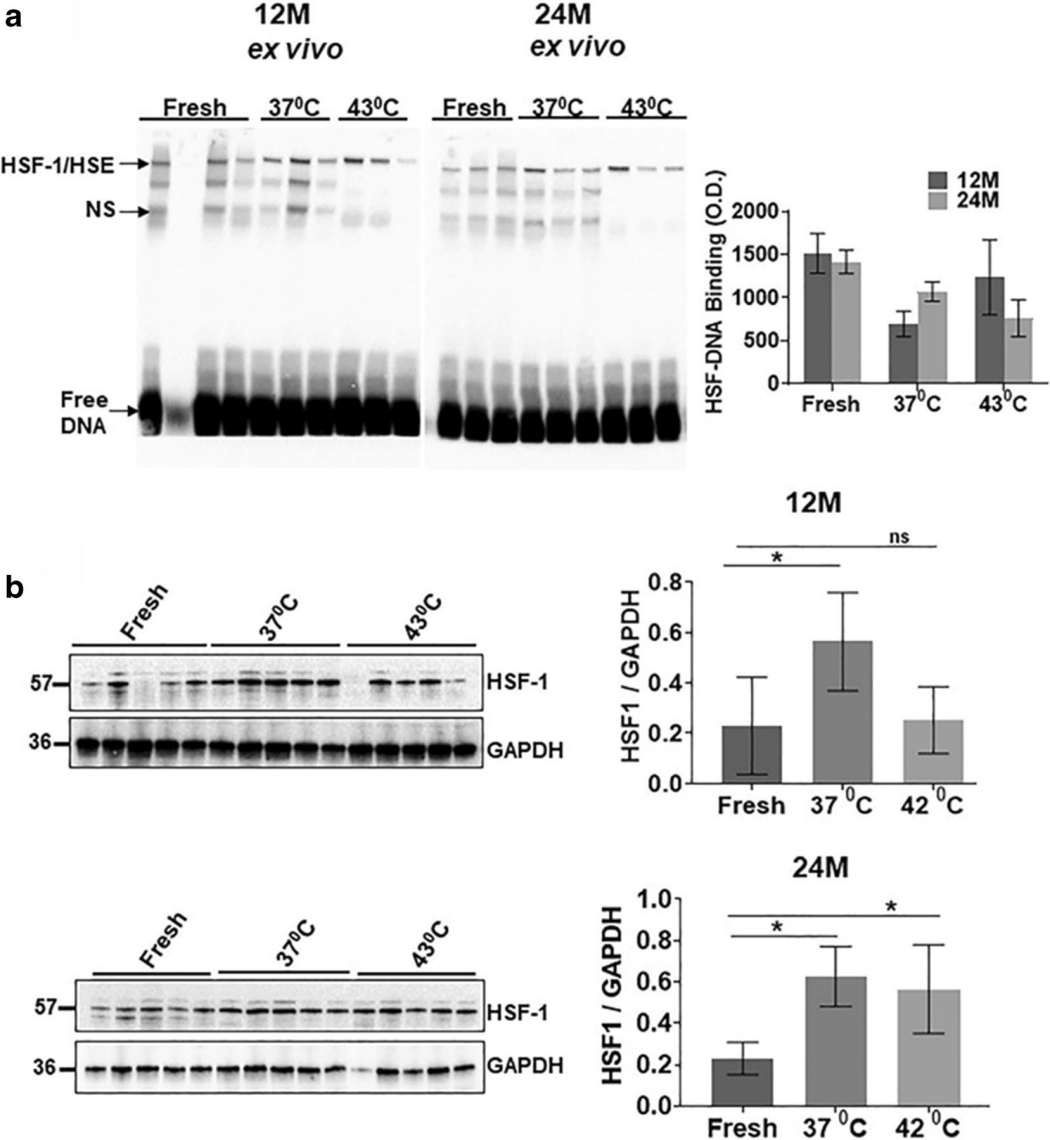
*Ex vivo* heat shock response in middle age compared to old Ames dwarf mice. **a** A representative EMSA of HSF1–HSE binding activity is shown for middle aged (12M) and old aged (24M) mouse liver that were either extracted freshly or cultivated *ex vivo* at 37 °C or 43 °C for 60 min. A bar graph shows HSF1–HSE binding levels from 12M and 24M liver samples taken from freshly sacrificed mice (fresh) and *ex vivo* liver samples incubated at 37 °C and 43 °C in oxygenated normal saline. Both 12M and 24M *ex vivo* liver samples display diminished HSF1–HSE binding at the recovery temperature of 37 °C when compared to freshly isolated liver, whereas heat-shocked *ex vivo* samples show mildly inducible HSF1–DNA binding only in 12M but not 24M samples. The heat inducible HSF1-HSE binding in *ex vivo* 12M samples was lower than freshly isolated liver tissue from both 12M and 24M old mice. Statistical analysis showed 12M fresh versus 24M freshly isolate liver with a *p* value = 0.9999; 12M freshly isolate liver versus 12M 37 °C ex vivo samples with a *p* value = 0.1889; 12M freshly isolated liver versus 24M *ex vivo* liver at 37 °C with a *p* value = 0.8276; 24M freshly isolated liver versus ex vivo liver at 43 °C with a *p* value = 0.4034. Thus, a 1-h “recovery period” of liver samples at 37 °C only partially attenuated HSF1 into its non-DNA binding state, and there was nominally inducible HSF1 DNA binding activity in the 12M but not 24M samples. **b** Western blot and densitometry graph analyses of HSF1 protein levels from 12M and 24M old dwarf mouse liver compares freshly isolated liver with *ex vivo* liver samples held at 37 °C or 43 °C for 1 h. A bar graph of the western blot analyses of HSF1 protein levels from *ex vivo* liver samples normalized against GADPH protein levels demonstrates a 2.5-fold increase in HSF1 protein levels when compared to freshly isolated liver samples. No age-dependent changes are noted in the *ex vivo* accumulation of HSF1 protein levels. All *p* values are *<* .05 with *p* = 0.0274 for 12M and *p* = 0.0045 for 24M when *ex vivo* liver samples are compared to freshly isolated liver samples.

With evidence supporting maintenance or enhancement of the heat shock axis in exceptional longevity [31, 45, 46], we investigated freshly isolated liver *ex vivo* for its response to a second stress from heat. Figure 3 a shows EMSA for HSF1–HSE binding in liver samples from 12M and 24M old liver perfused with either 37 °C or 43 °C warmed in normal saline that was oxygen perfused for 1 h. The bar graph shows that *ex vivo* manipulation of the liver samples results in lower levels of HSF1–HSE binding in both 12M and 24M at 37 °C relative to freshly isolated samples. When the liver samples were heat shocked at 43 °C, HSF1–HSE binding levels were lower relative to freshly isolated liver samples from both 12M and 24M Ames dwarf mice. Apoptosis markers were not elevated in the *ex vivo* samples (data not shown). The 24M liver samples from dwarf mice showed a non-significant trend towards lower HSF1–HSE binding levels. Notably, despite the decline in HSF1–HSE binding relative to freshly isolated liver, 12M old livers can mount a second, stress-inducible increase in HSF1–HSE binding activity, whereas the 24M *ex vivo* liver samples cannot bolster their HSF1–HSE binding activity during heat stress.

To determine if an *ex vivo* decline in HSF1–HSE binding was due to loss of HSF1 levels, we measured HSF1 protein by western blots. Contrary to expectations, HSF1 protein levels increased by over 2-fold in both 12M and 24M *ex vivo* liver samples relative to the freshly isolated samples (Fig. 3b). Furthermore, the 24M old *ex vivo* tissue sustained HSF1 protein levels during heat stress, whereas the 12M old tissue did not.

## Discussion

As a model of exceptional longevity, Ames dwarf mice show robust life spans when compared to their wild type counterparts [47, 48]. Ames dwarf mice show spontaneous recessive loss-of-function mutation in the prophet of pituitary factor-1 (*Prop-1*) gene that promotes a dramatic decline in circulating growth hormone (GH), prolactin (PRL), and thyroid stimulating hormone (TSH) [49]. Diminished insulin/IGF-1 signaling plays an important role in promoting longevity and stress resistance in dwarf mice [50]. Extended longevity in the Ames dwarf mouse model depends on peripubertal GH status and on average, their lifespan is increased by over 49% in males and 68% in females [51]. Recent reports show that Ames dwarf mice resist the lethal effects of oxidative toxins diquat and paraquat in vivo [47]. This observation coupled with vigorous stress resistance in long-lived poikilotherms, suggests a critical role of the heat shock axis in regulating exceptional longevity [52, 53]. Having probed both positive and negative regulatory checkpoints of the heat shock axis in dwarf mice, the bulk of evidence suggests that exceptional longevity is linked to maintenance of the heat shock response with enhancement or age-dependent compensation of several HSF1 regulatory checkpoints in favor of a robust heat shock axis [45, 54, 55].

### Role of stress-induced protein–HSF1 interactions

One clear advantage observed in old (24M) dwarf mice is their ability to reduce the pool of proteins known to inhibit HSF1. One mechanism to attenuate the HSR entails complexes of HSP70, HSP40. and HSBP1 that convert HSF1 trimer to an inert monomer [56]. With age, Ames dwarf mice exhibit lower levels of HSP70, HSP90, and HSP40 that make up the multiprotein complex that normally downregulates HSF1–promoter binding. Similarly, aged Ames dwarf mice express lower levels of heat shock factor binding protein 1 (HSBP1) which otherwise would interfere with HSF1 trimerization during stress. The concomitant elevation of HSF1 protein levels with dwarf aging further substantiate a heat shock response well poised with high levels of HSF1 and low levels of its inhibitory proteins, thus creating conditions highly favorable to heat shock axis activation. Results from our study shows that liver from aged Ames dwarf mice exhibit nearly 30% more HSF1 protein and up to 30% less heat shock proteins (HSP40, HSP70, and HSP90) than middle-aged mice. The results in this report need to be evaluated relative to other studies that examine the role of life—extending endocrinological changes upon the heat shock axis. Long lived *C. elegans* with knockout GH/IGF1 exhibit elevated HSP expression [57]. Similarly, long-lived mice with diminished GH/IGF-I signaling exhibit heart tissue elevation of three HSP mRNAs (Hsf2, ,Hsp90aa, and Dnajc3) and four liver HSP mRNAs (Hspb7, Hspa9, Hsp47 and Hsf4) [58]. All these observations represent comparisons between young or mature mice GH variants with their wild type counterpart; thus, it is unknown whether elevated expression of HSP or their mRNA carries over into senescent animals as we have found in this report. We also parenthetically note that HSP and its mRNA expression vary between different tissue samples in long-lived mice with disrupted GH/IGF-1 pathways. For example, instances where liver HSP are elevated in long-lived mice their kidneys may demonstrate lower levels of HSP [58]. Notably, none of these studies examines the *inducibility* of heat shock genes in long-lived mice relative to their wild type counterparts, and certainly none of the studies ascertain the role of GH/IGF1 axis in conferring senescent-enhanced heat shock responsivity as we report here.

The observations from our study suggest that exceptional longevity is associated with higher levels of HSF1 and lower levels of factors meant to squelch HSF1 activity. The longevity effect on HSF1 appears to be specific to this transcription factor, as another member of the HS transcription factor family such as HSF2 does not increase in aging dwarf mice.

### Role of HSF1 post-translational changes

Further evidence for longevity being associated with upregulation of activating pathways and suppression of deactivating pathways during stress is apparent from our analysis of HSF1 post-translational modifications [30, 59]. For example, HSF1 acetylation in the DNA-binding domain negatively regulates HSF1 binding to the heat shock element. Here we find three isoforms of HSF1 with *decreased* acetylation in aging dwarf mice. This contrasts sharply with aging wild-type mice that exhibit over 2-fold increase in HSF1 acetylation levels [30]. Given our observation of elevated SIRT1 levels with aging dwarf mice, we speculate that preservation of this enzymatic activity with age is a key factor in preserving the heat shock axis as a mechanism for exceptional longevity. The critical role of SIRT1 in supporting longevity is further supported by the observation that protein levels of this deacetylase decline with aging wild type Ames mice [30].

Another means of regulating the heat shock axis is through differential phosphorylation [29, 60]. While bulk HSF1 phosphorylation is thought to enhance its promoter functionality, specific amino acid residues such as Ser326 enhance HSF1 transcriptional activity in response to heat stress [61–63]. Aging usually diminishes stress inducible Ser326 phosphorylation, whereas aging dwarf mice preserve Ser326 phosphorylation levels with non-statistical trends towards enhanced phosphorylation of HSF1 at Ser326. Conversely, HSF1 phosphorylation on Ser303 and Ser307 inhibit HSF1 transcriptional activity under normal physiological conditions, most likely affecting protein-protein interactions within transcriptional complexes [24, 64]. Our results demonstrate an age-associated decrease in dwarf HSF1 phosphorylation on Ser307 by approximately 25% when normalized against total HSF1. Thus, the pattern of HSF1 in aging dwarf mice supports the notion that exceptional longevity is associated with enhancement of stimulatory pathways for HSF1 and attenuation of the inhibitory phosphorylation-linked signaling pathways. Due to multiple stress inducible kinases converging on HSF1 [65–67], it is not clear if one or several signaling systems are responsible for ramping up the stress response during exceptional longevity. In a parallel manner, exceptional longevity may also be associated with changes in phosphatase activity required for Ser307 dephosphorylation [59].

### Repetitive stress and longevity

Given the perspective that exceptional longevity results in a heightened responsivity to stress [47, 68, 69], a plausible thought is that aging dwarf mice would aptly handle repetitive stress. An *ex vivo* model of organ stress was used to explore how exceptionally long-living mice handled repetitive stress. A surprising result of these experiments was the fact that HSF1 protein levels increased by nearly threefold after 1 h of *ex vivo* perfusion of liver with 37 °C normal saline in both 12M and 24M dwarf mice. To our knowledge, this is the first evidence that HSF1 protein levels are upregulated during the stress recovery period, perhaps preparing cells for another wave of stress. Upon heat shock, the aging dwarf mice were able to sustain HSF1 protein levels, albeit there was a downward trend suggesting that aging dwarf mice, like their wild type counterparts, may be more susceptible than their younger counterparts to repetitive stress.

## Summary

**Fig. 4:**
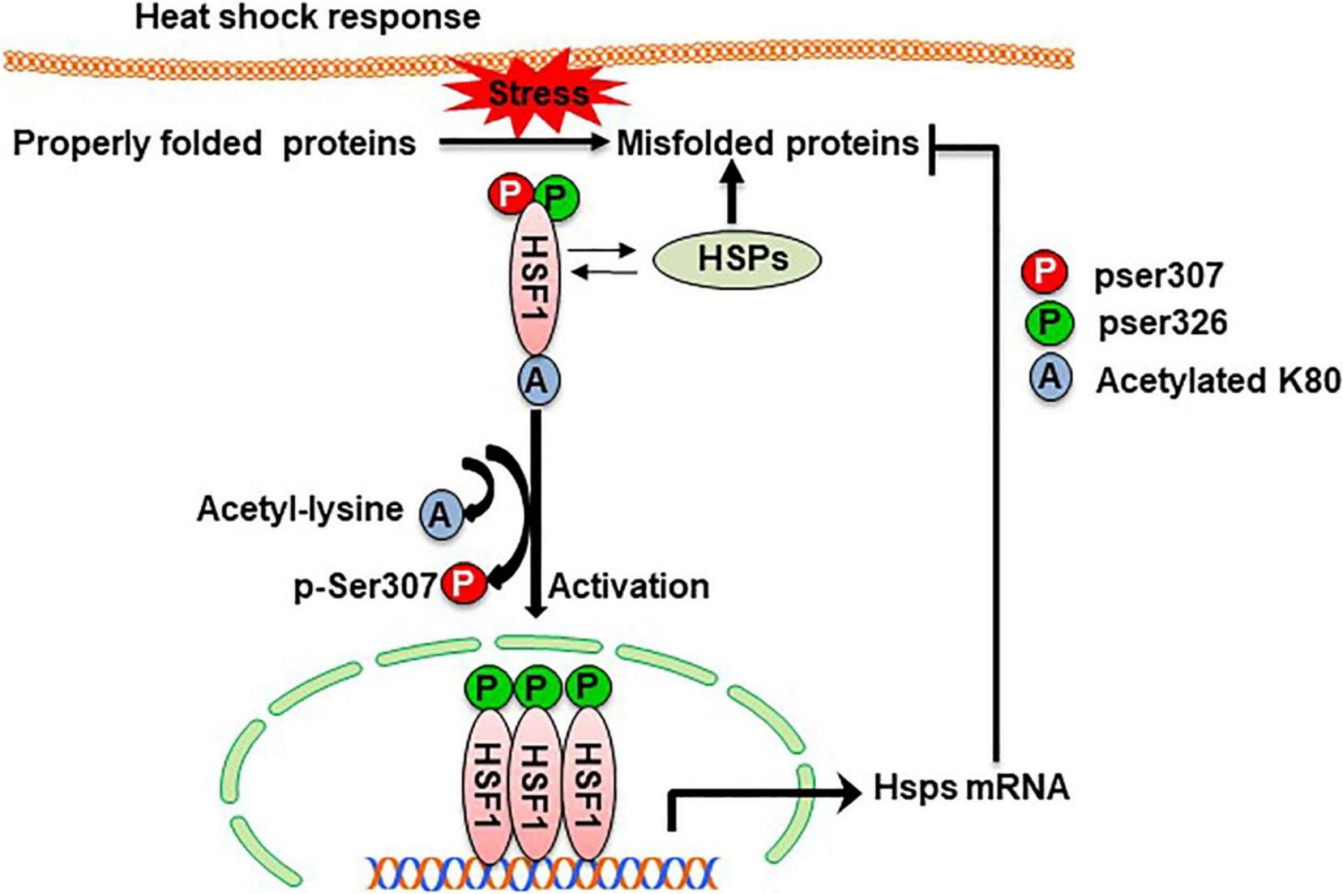
A model of how the heat shock axis is preserved in long-lived Ames mice. Overall, the model summarizes data from this report that supports the hypothesis that exceptional longevity is associated with enhanced activating pathways and diminished inhibitory pathways that regulate HSF1. Figure key: HSF1, heat shock factor 1; HSP, heat shock protein; mRNA, messenger RNA

This study reveals that old, long-lived Ames dwarf mice exhibit a robust heat shock response associated with amplified activating pathways and attenuated deactivating pathways (see Fig. 4). Heat shock enhancements appear to be age-inducible changes in dwarf mice rather than preservation of a superior heat shock response in middle-aged dwarf relative to wild-type mice. In some respects, our results suggest that exceptional longevity requires over compensation of age acquired elements that thwart the heat shock response. These compensatory changes include disruption of HSF1–protein interactions and inhibitory post-translational modification of HSF1. As a cross-species comparison, long-lived naked mole rats similarly express low heat shock protein levels in whole cell extracts when compared to dwarf mice [70, 71]. This comparative finding highlights the potential significance of declining basal heat shock protein levels with age. Less certain is whether longevity depends upon HSP redistribution to different cellular compartments as suggested in the naked mole experiments. Longevity also appears to require preserved or enhanced SIRT1 protein levels or activity [8, 72, 73]. Whether longevity needs enhanced intracellular signals that converge on the heat shock axis remains a possibility. The fact that exceptional longevity is associated with post-stress accumulation of HSF1 is intriguing and suggests a potential therapeutic avenue for age–related conditions such as Alzheimer’s disease which is known to have low levels of HSF1 [59, 74, 75].

While longevity appears to be associated with a heightened heat shock response [31, 45, 76], there are some limitations to our observations. Firstly, we do not know if our observations are applicable to human exceptional longevity. Secondly, we do not know if prolonged stress as opposed to repetitive stress is handled differently in long-lived mice. Thirdly, we do not see major differences between middle-aged dwarf and wild-type mice that would predict the age-dependent enhancement of the heat shock response in the dwarf mice. Fourthly, we did not find significant differences in the HS response with gender that might account for gender differences seen with dwarf longevity. Fifthly, we do not know how low levels of HSBP1 and HSPs are achieved in long-living Ames dwarf mice given that aging is not associated with a generalized decrease in protein translation. One interpretation of our findings may be that low levels of protein aggregates in long-lived Ames dwarf mice compared to Ames wild--type mice somehow downregulate mRNA for HSPs and HBPI. Also noted is an age-associated increase in protein ubiquitination reported in long-lived naked mole rats that may further suppress HSPs and HBPI protein levels [77, 78]. Of note, this study conducted a cross sectional analysis of middle aged and old mice and a limitation is that changes over the complete Ames dwarf mice lifespan are not fully known, for instance, with 3, 6, and 30-month-old mice. It would have been better to include more intermediate age groups to get a clear picture of age-dependent changes. Ideally, a longitudinal study of the same mice over time would reveal this mouse strain’s transition to a more robust stress response with age.

Finally, given the cross sectional study design, we do not know the exact age when dwarf mice begin to bolster their heat shock responsivity after 12 months. Nonetheless, our results support a hypothesis that *attenuation* of HSF1 deactivating signals and *enhancement* of its activating signals can promote exceptional longevity (see Table 2 for a summary of wild type versus dwarf mice differences in the heat shock response).

**Table 2:** HSR changes with age in Ames wild type and dwarf mice. Overall, the table shows that with each component of HSF1 regulation, Ames dwarf, unlike the wild type, manifest heighted activation signals and lower inhibitory signals that control HSF1 transcriptional activity

	Ames wild type[30]	Ames dwarf
Age-dependent changes in heat shock axis		
HSF1–HSE binding activity	55% decline	10% decline
HSF1 protein level	48% decline	35% increase
Inhibitory and activating HSF1 phosphorylation normalized against HSF1		
Ser326	58% decrease	No change
Ser307	No change	25% decrease
HSF1 acetylation status		
HSF1 acetylation on lysine 80	2.9 fold increase	6-fold decrease
SIRT1 protein levels	30% decline	36% increase
HSF1 mRNA level	30% decline	3-fold increase
Expression of proteins that negatively regulate HSF1		
HSP90	19% increase	27% decline
HSP70	23% increase	30% decline
HSP40	40% increase	37% decline
HSBP1	27% increase	38% decline

## Data Availability

Not applicable
